# Supplementary material for: Unraveling DPP4 Receptor Interactions with SARS-CoV-2 Variants and MERS-CoV: Insights into Pulmonary Disorders via Immunoinformatics and Molecular Dynamics
Source: Viruses. 2023 Oct 6;15(10):2056. doi: 10.3390/v15102056 (PMC10612102; doi:10.3390/v15102056)
Supplement: Supplementary file 1 [file viruses-15-02056-s001.zip › viruses-2629526-supplementary file.pdf]

Table S1: List of mutations for each variant of SARS CoV-2. The crucial mutations are marked in bold.

SARS CoV-2 Variants	Mutation
Alpha (B.1.1.7)	69del, 70del, 144del, V70L, D178H, **E484K**, N501Y, P681H, A570D
Gamma (P.1)	N501Y, K417T, **E484K**
Beta (B.1.351)	N501Y, K417N, E484K, L18F, D80A, D215G, R246I, A701V
Delta (B.1.617.2)	**L452R**, **T478K**, G142D, 156del, 157del, R158G, D614G, P681R, D950N
Omicron (B.1.1.529)	K417N, N440K, G446S, S477N, T478K, E484A, Q493K, G496S, Q498R, N501Y, Y505H

Table S2: The key residues forming the binding partner between spike protein of Wildtype SARS-CoV-2 and DPP4

DPP4 residue	SARS-CoV-2 Wildtype residue	Mode of interaction	Frequency (%)
A282	V483	Hydrophobic	40
A289	Q493	Hydrogen bond	40
A291	Q493	Hydrogen bond	32
F269	V483	Hydrophobic	51.6
I285	V483	Hydrophobic	49.1
A291	Y495	Hydrogen bond	55.5
A342	N448	Hydrogen bond	56.7
Q286	E484	Hydrogen bond	48.3
D331	S494	Hydrogen bond	49.6
R336	E484	Salt bridge	100
K392	D442	Salt bridge	100
D393	K444	Salt bridge	100
R343	D442	Salt bridge	100

Table S3: The key residues forming the binding partner between spike protein of Alpha SARS-CoV-2 and DPP4

DPP4 residue	SARS-CoV-2 Alpha residue	Mode of interaction	Frequency (%)
A282	V483	Hydrophobic	43.45
A289	Q493	Hydrogen bond	49.32
F269	V483	Hydrogen bond	65.87
I285	V483	Hydrophobic	80.57
I287	V483	Hydrophobic	71.37
Q344	K444	Hydrophobic	34.11
K392	D442	Salt bridge	100
R336	E471	Salt bridge	100
S284	K484	Hydrogen bond	40.70
Q286	K484	Hydrogen bond	42.01
A342	N450	Hydrogen bond	37.69

Table S4: The key residues forming the binding partner between spike protein of Beta SARS CoV-2 and DPP4

DPP4 residue	SARS-CoV-2 Beta residue	Mode of interaction	Frequency (%)
A289	Q493	Hydrogen bond	33.90
A291	F456	Hydrophobic	51
A291	Q493	Hydrophobic	58.73
I285	V483	Hydrophobic	20.21
E347	K444	Salt bridge	100
E378	K444	Salt bridge	100
D393	K444	Salt bridge	100
A291	Y495	Hydrogen bond	67.44
L294	G502	Hydrogen bond	37.70
S292	G504	Hydrogen bond	37.16

Table S5: The key residues forming the binding partner between spike protein of Delta SARS-CoV-2 and DPP4

DPP4 residue	SARS-CoV-2 Delta residue	Mode of interaction	Frequency (%)
A282	V483	Hydrogen bond	45.68
A289	Q493	Hydrophobic	43.69
A291	Q493	Hydrophobic	43.41
K287	T478	Hydrophobic	62.86
D331	R452	Salt bridge	100
K392	D442	Salt bridge	100
E191	K478	Salt bridge	100
R336	E471	Salt bridge	100
K190	E484	Salt bridge	100
E347	K444	Salt bridge	100
E332	R452	Salt bridge	100
D390	K444	Salt bridge	100
D192	K478	Salt bridge	100
R343	D442	Salt bridge	100
S284	C488	Hydrogen bond	53.75
S284	N487	Hydrogen bond	47.52
S284	G485	Hydrogen bond	46.53
Q344	G504	Hydrogen bond	44.10
Y225	N487	Hydrogen bond	37.35
E191	S477	Hydrogen bond	37.17

Table S6: The key residues forming the binding partner between spike protein of Gamma SARS-CoV-2 and DPP4

DPP4 residue	SARS-CoV-2 Gamma residue	Mode of interaction	Frequency (%)
A282	V483	Hydrophobic	40.89
A289	Q493	Hydrogen bond	50.56
A291	Q493	Hydrophobic	44.60
F269	V483	Hydrophobic	34.37
I285	V483	Hydrogen bond	21.11
I287	V483	Hydrophobic	29.40
Q286	N481	Hydrophobic	27.15
Q344	K444	Hydrophobic	32.69
E332	K484	Salt bridge	100
D393	K444	Salt bridge	100
A342	N448	Hydrogen bond	36.50

Table S7: The key residues forming the binding partner between SARS-CoV-2 Omicron and DPP4

DPP4 residue	SARS-CoV-2 Omicron residue	Mode of interaction	Frequency (%)
A282	V483	Hydrophobic	40.45
A289	Q493	Hydrophobic	51.26
Q344	K444	Hydrophobic	24.98
T288	Y505	Hydrophobic	53.71
D390	K444	Salt bridge	100
N281	C488	Hydrogen bond	51.65
A282	N487	Hydrogen bond	28.88

Figure legends

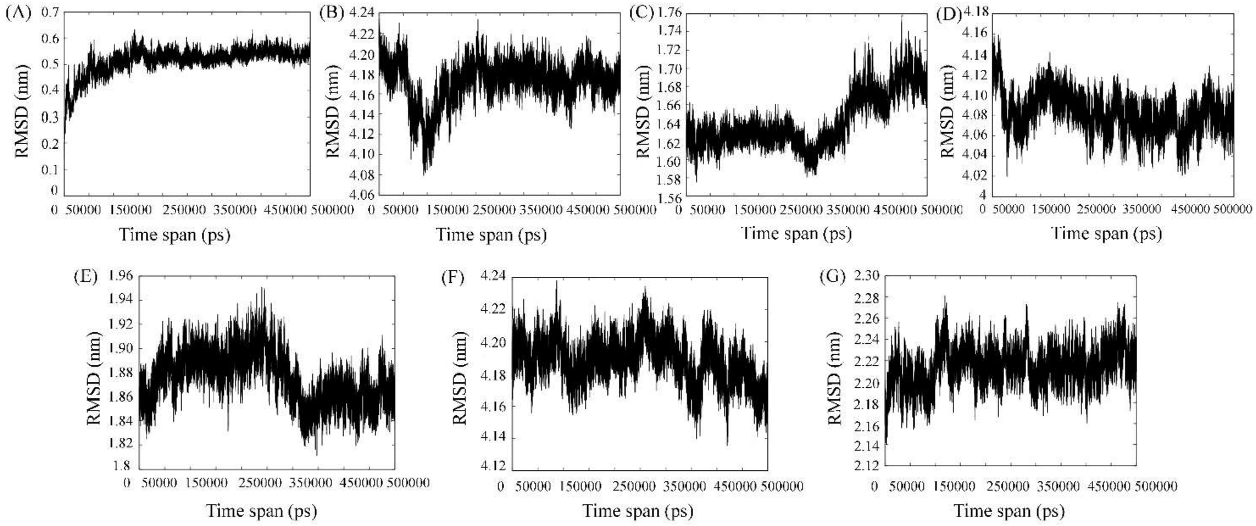

Figure S1: RMSD plot of (A) MERS CoV:DPP4, (B) wild-type SARS CoV-2:DPP4, (C) Alpha SARS CoV-2:DPP4, (D) Beta SARS CoV-2:DPP4, (E) Delta SARS CoV-2:DPP4, (F) Gamma-SARS CoV-2:DPP4, (G) Omicron SARS CoV-2:DPP4.

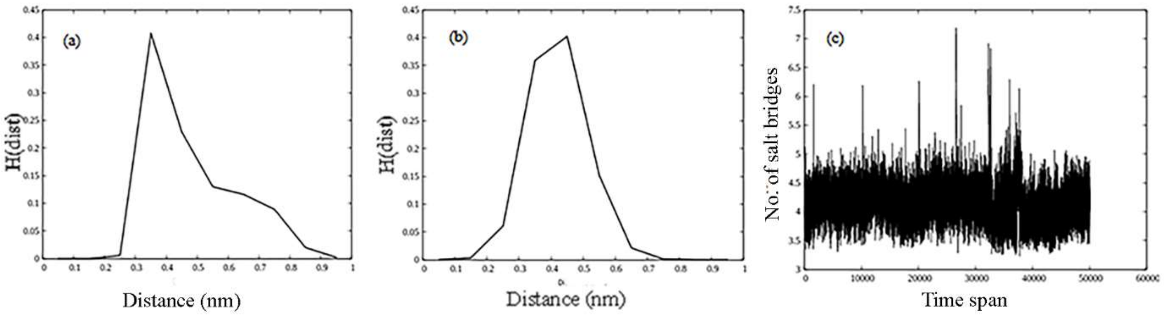

Figure S2: (A) Histogram plot of hydrophobic interaction between A282(DPP4) and V483(wild-type SARS CoV-2), (B) Histogram plot of hydrogen interaction between A289(DPP4) and Q493(wild-type SARS CoV-2), (C) Number of Salt bridge interaction between K392(DPP4) and D442(wild-type SARS CoV-2).

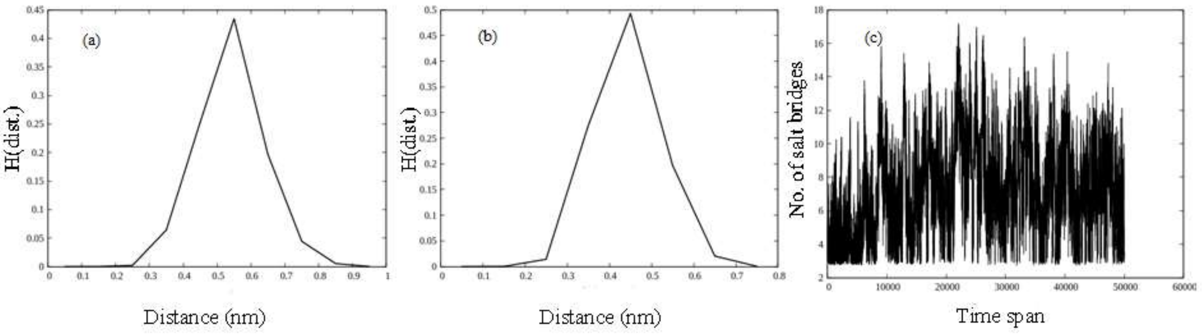

Figure S3: (A) Histogram plot of hydrophobic interaction between A282(DPP4) and V483(alpha SARS CoV-2), (B) Histogram plot of hydrogen bond interaction between A289(DPP4) and Q493(alpha SARS CoV-2), (C) Number of Salt bridge interaction between R336(DPP4) and E484(alpha SARS CoV-2).

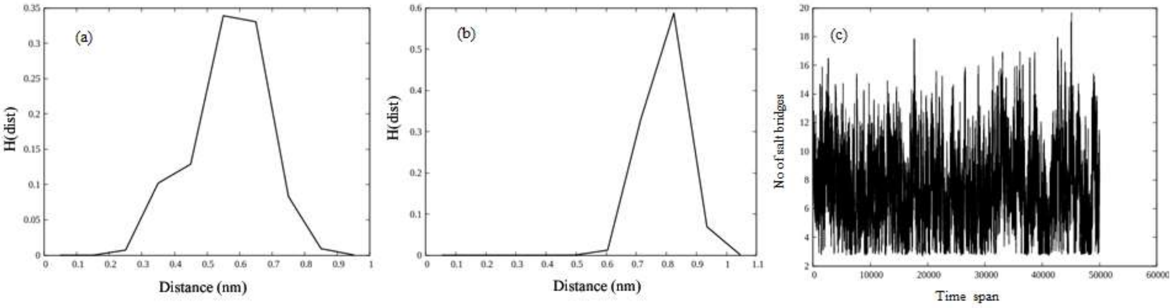

Figure S4: (A) Histogram plot of hydrogen bond interaction between A289(DPP4) and Q493(beta SARS CoV-2), (B) Histogram plot of hydrophobic interaction between A291(DPP4) and Q493(beta SARS CoV-2), (C) Number of Salt bridge interaction between D393(DPP4) and K444(beta SARS CoV-2).

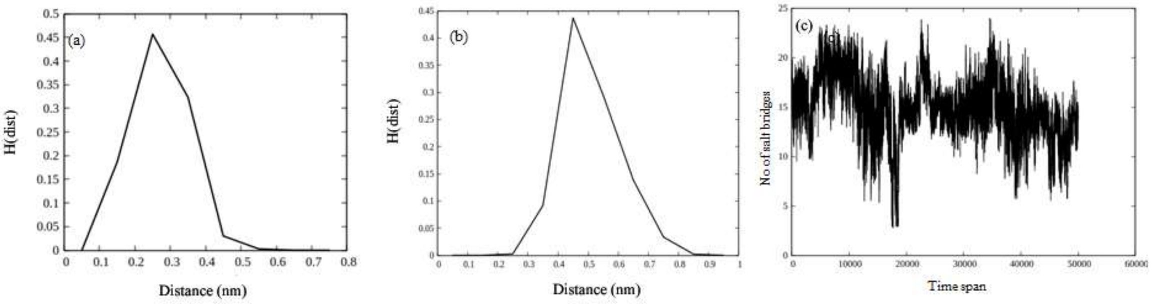

Figure S5: (A) Histogram plot of hydrophobic interaction between A282(DPP4) and V483(delta SARS CoV-2), (B) Histogram plot of hydrogen bond interaction between A289(DPP4) and Q493(delta SARS CoV-2), (C) Number of Salt bridge interaction between D390(DPP4) and K444(delta SARS CoV-2).

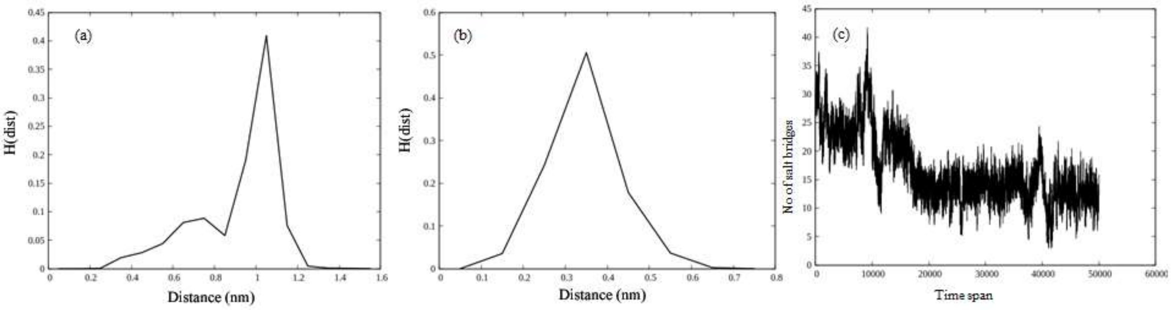

Figure S6: (A) Histogram plot of hydrophobic interaction between A282(DPP4) and V483(gamma SARS CoV-2), (B) Histogram plot of hydrogen bond interaction between

A289(DPP4) and Q493(gamma SARS CoV-2), (C) Number of Salt bridge interaction between E332(DPP4) and K484(gamma SARS CoV-2).

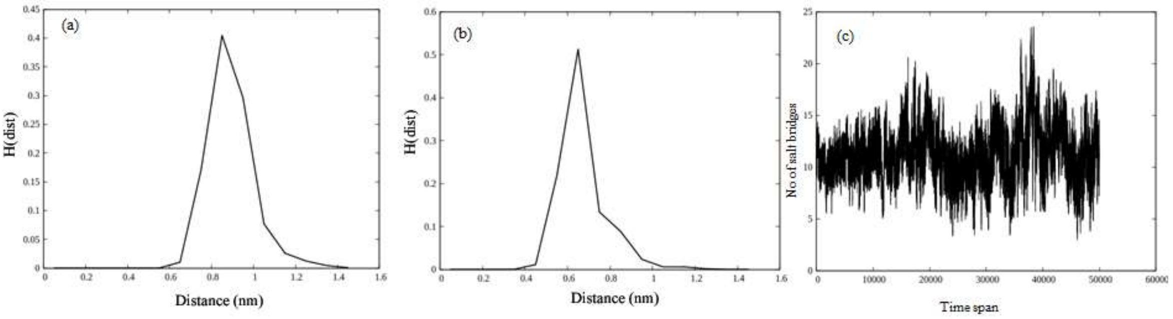

Figure S7: (A) Histogram plot of hydrophobic interaction between A282(DPP4) and V483(omicron SARS CoV-2), (B) Histogram plot of hydrophobic interaction between A289(DPP4) and Q493(omicron SARS CoV-2), (C) Number of Salt bridge interaction between D390(DPP4) and K444(omicron SARS CoV-2).

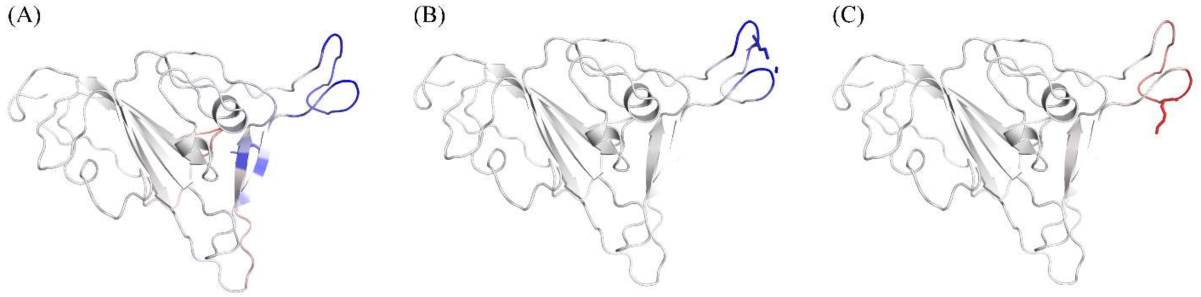

Figure S8: Visual representation of the interface mutations on dynamicity and plasticity of the RBD. RBD structure with the (A) L452R and (B) T478K mutation of Delta variant. RBD structure with the E484K mutation of (C) Gamma and Alpha variant. The RBD is represented as cartoon structure and mutations are shown as stick model. Red color in the cartoon structure indicates the flexibility in the protein and blue represents a rigidification of the structure. RBD, receptor binding domain

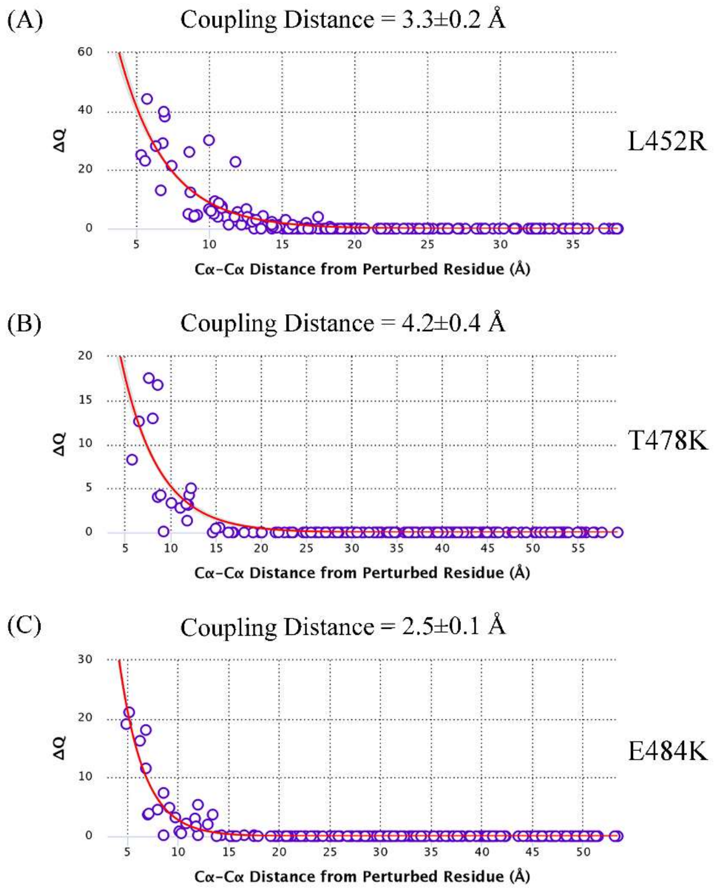

Figure S9: Distance dependence of the magnitude of perturbation on other residues (ΔQ) from the perturbed site of (A) R452 & (B) K478 in Delta and (C) K484 in Gamma and Alpha

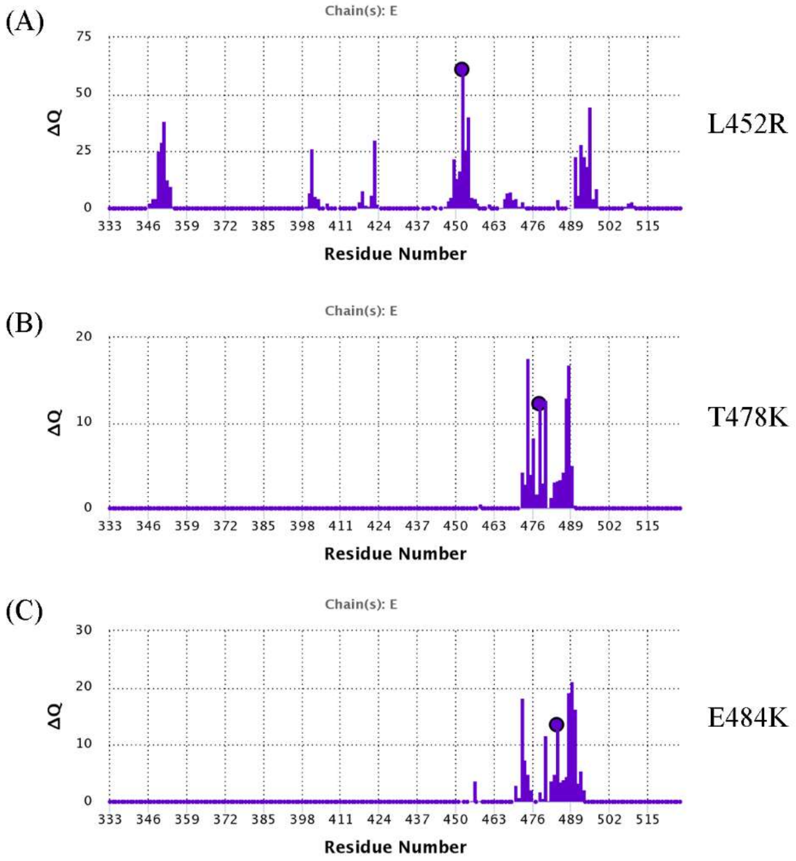

Figure S10: Residue-wise Perturbation Residue Scanning profile (ΔQ) distance from the perturb site which is shown in dot in the graph shown for three interface mutations present on the RBD. (A) L452R (B) T478K (C) E484K (the residue that is perturbed is highlighted by a circle).
