# Supplementary figures and images for: Unraveling DPP4 Receptor Interactions with SARS-CoV-2 Variants and MERS-CoV: Insights into Pulmonary Disorders via Immunoinformatics and Molecular Dynamics
Source: Viruses. 2023 Oct 6;15(10):2056. doi: 10.3390/v15102056 (PMC10612102; doi:10.3390/v15102056)

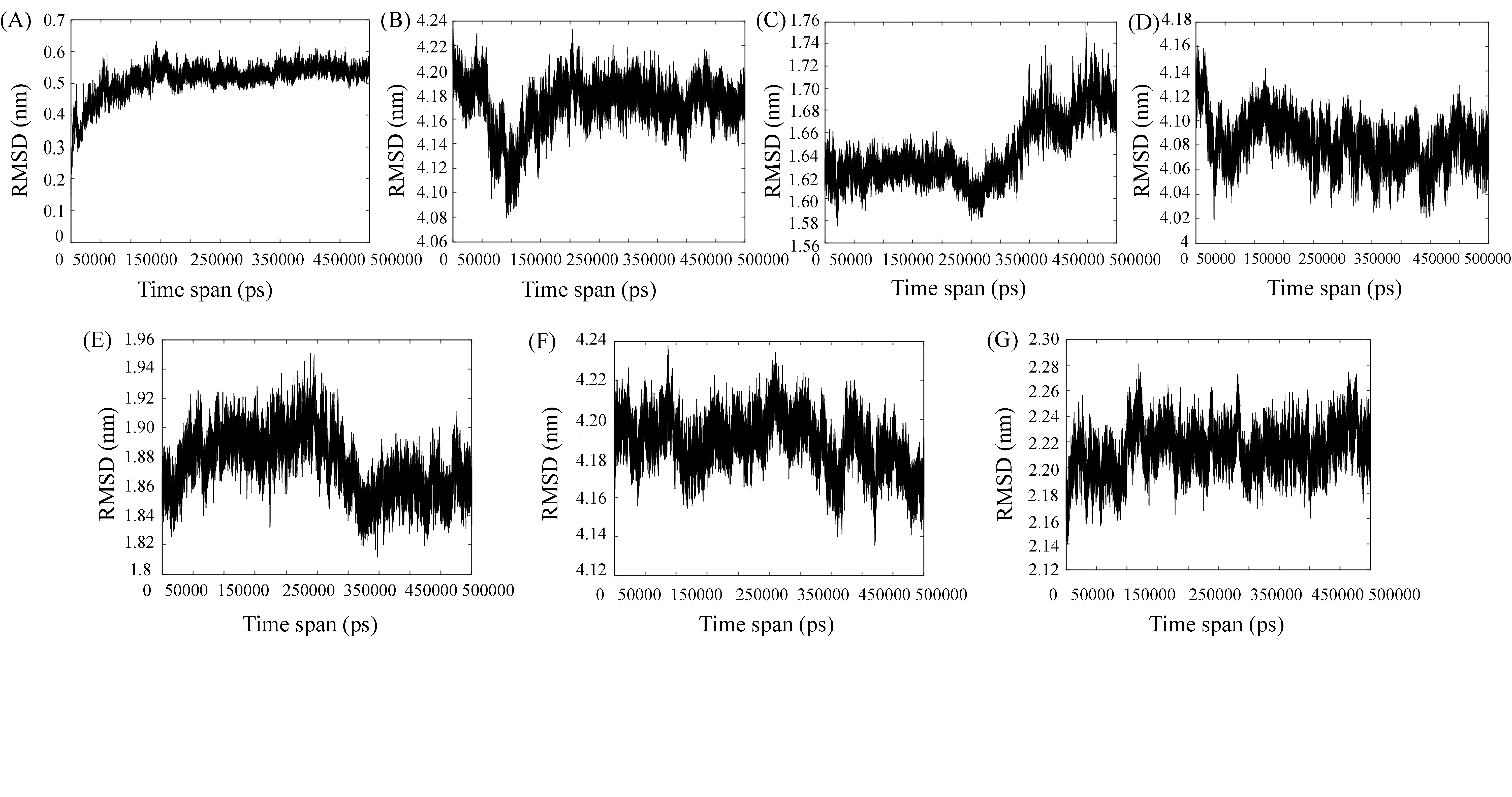

Supplement: Supplementary file 1 [file viruses-15-02056-s001.zip › Figure S1.tif]

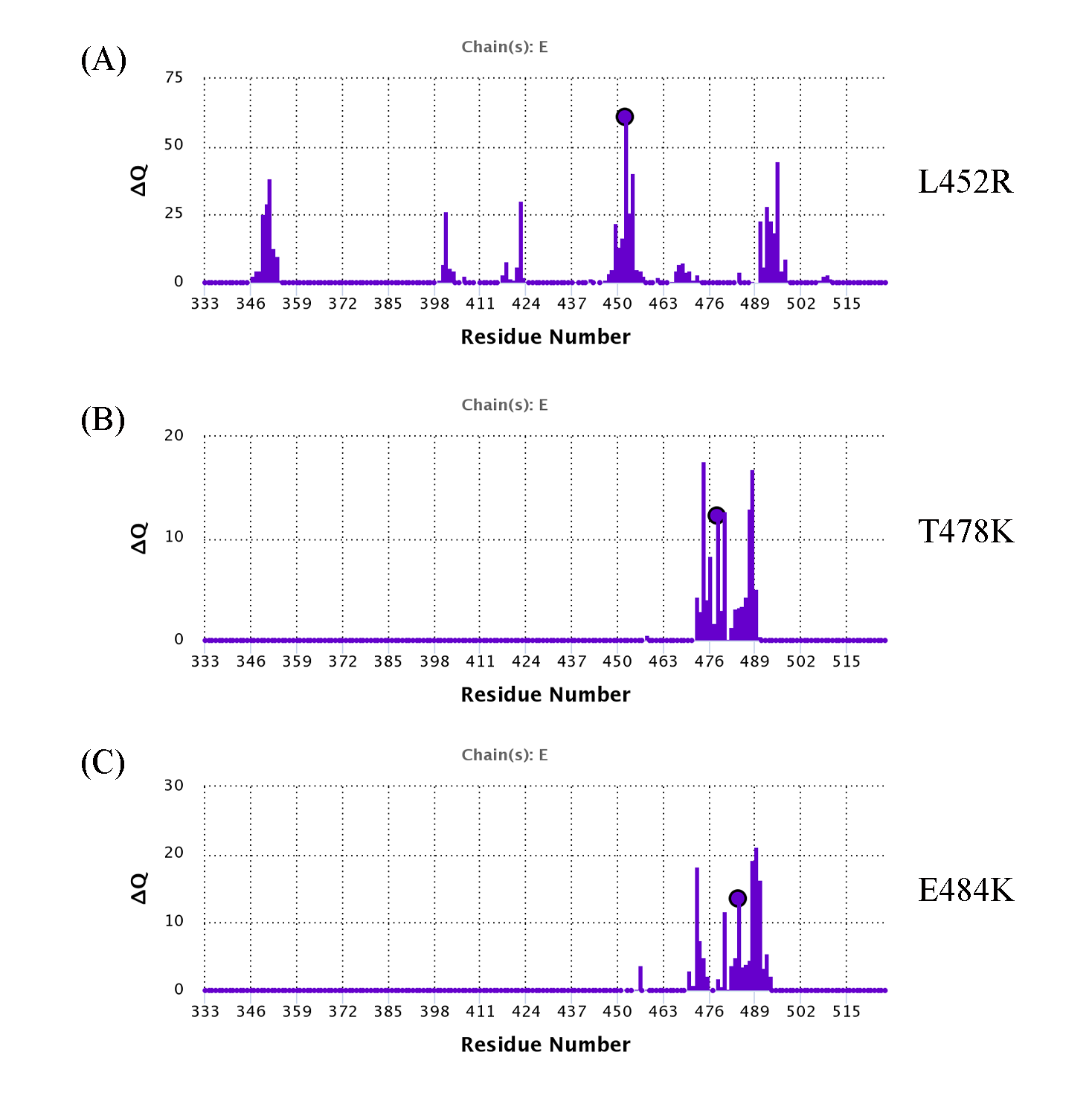

Supplement: Supplementary file 1 [file viruses-15-02056-s001.zip › Figure S10.tif]

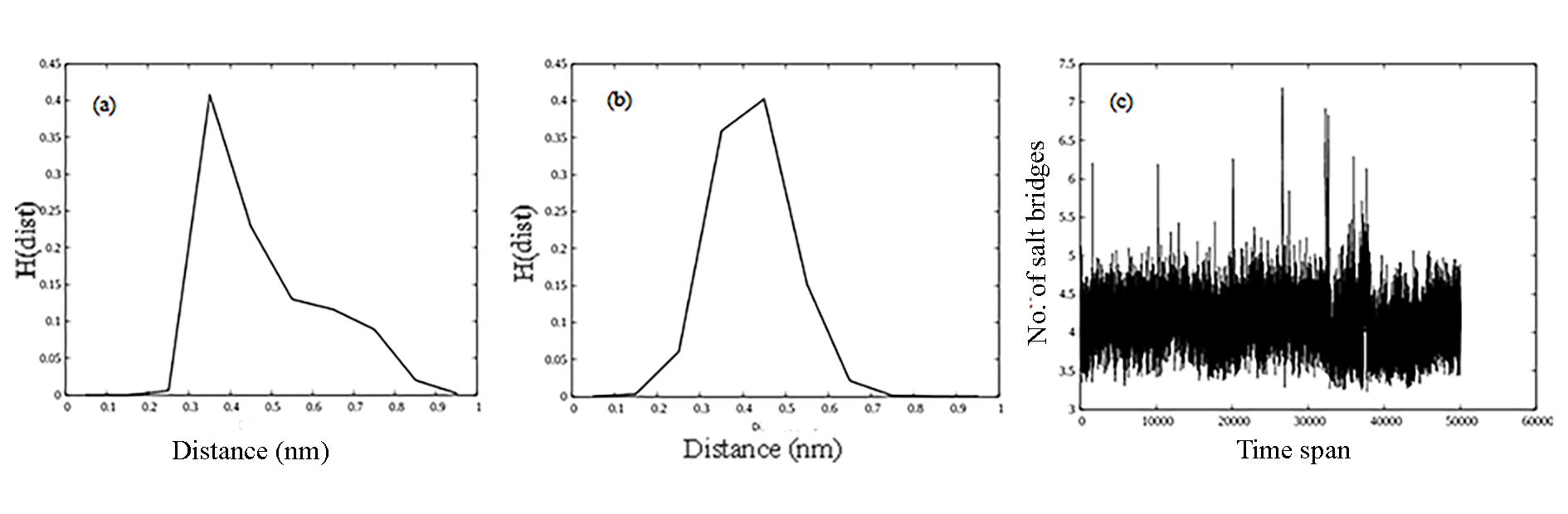

Supplement: Supplementary file 1 [file viruses-15-02056-s001.zip › Figure S2.tif]

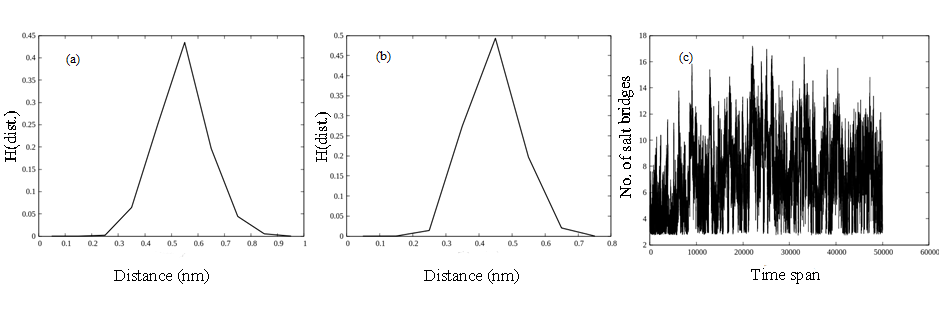

Supplement: Supplementary file 1 [file viruses-15-02056-s001.zip › Figure S3.tif]

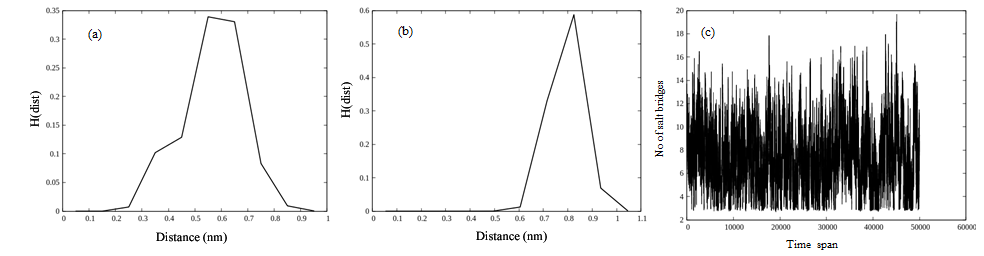

Supplement: Supplementary file 1 [file viruses-15-02056-s001.zip › Figure S4.tif]

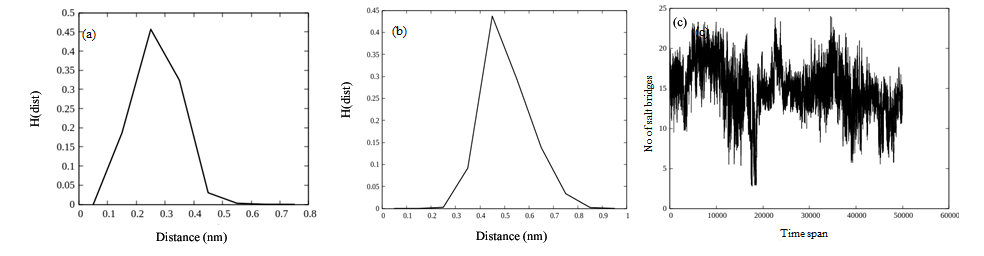

Supplement: Supplementary file 1 [file viruses-15-02056-s001.zip › Figure S5.tif]

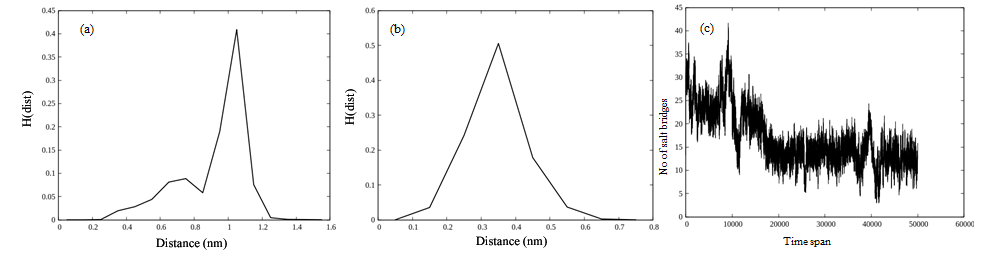

Supplement: Supplementary file 1 [file viruses-15-02056-s001.zip › Figure S6.tif]

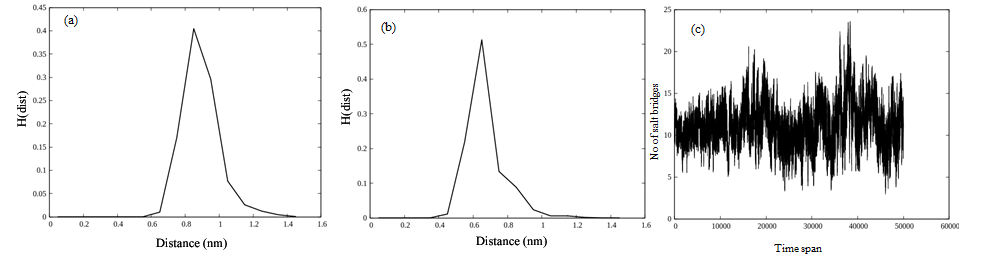

Supplement: Supplementary file 1 [file viruses-15-02056-s001.zip › Figure S7.tif]

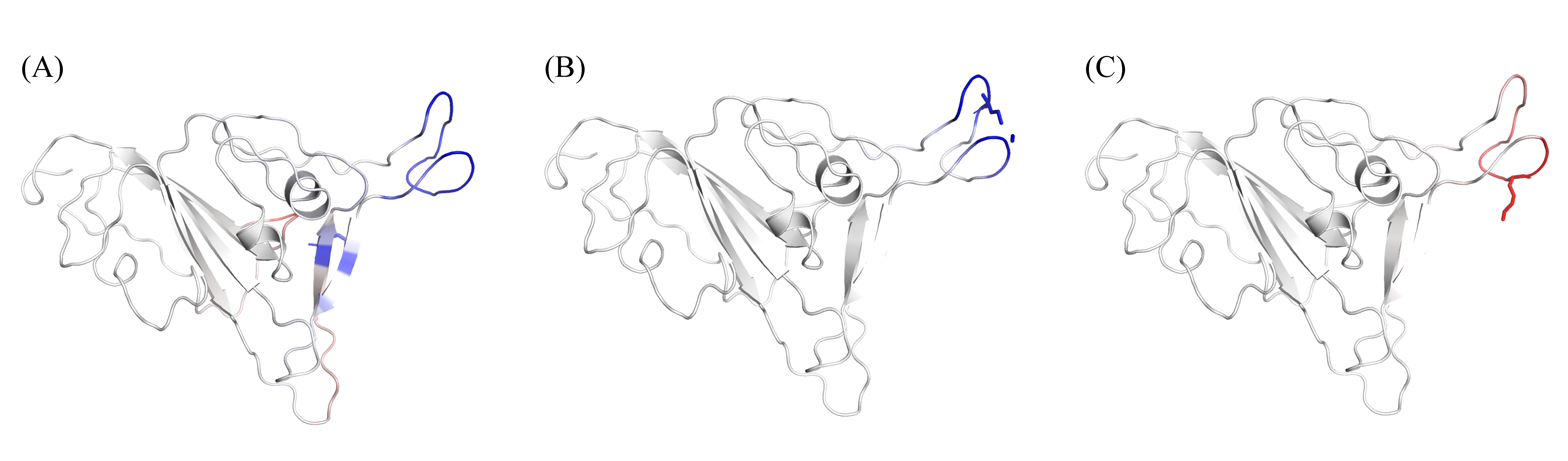

Supplement: Supplementary file 1 [file viruses-15-02056-s001.zip › Figure S8.tif]

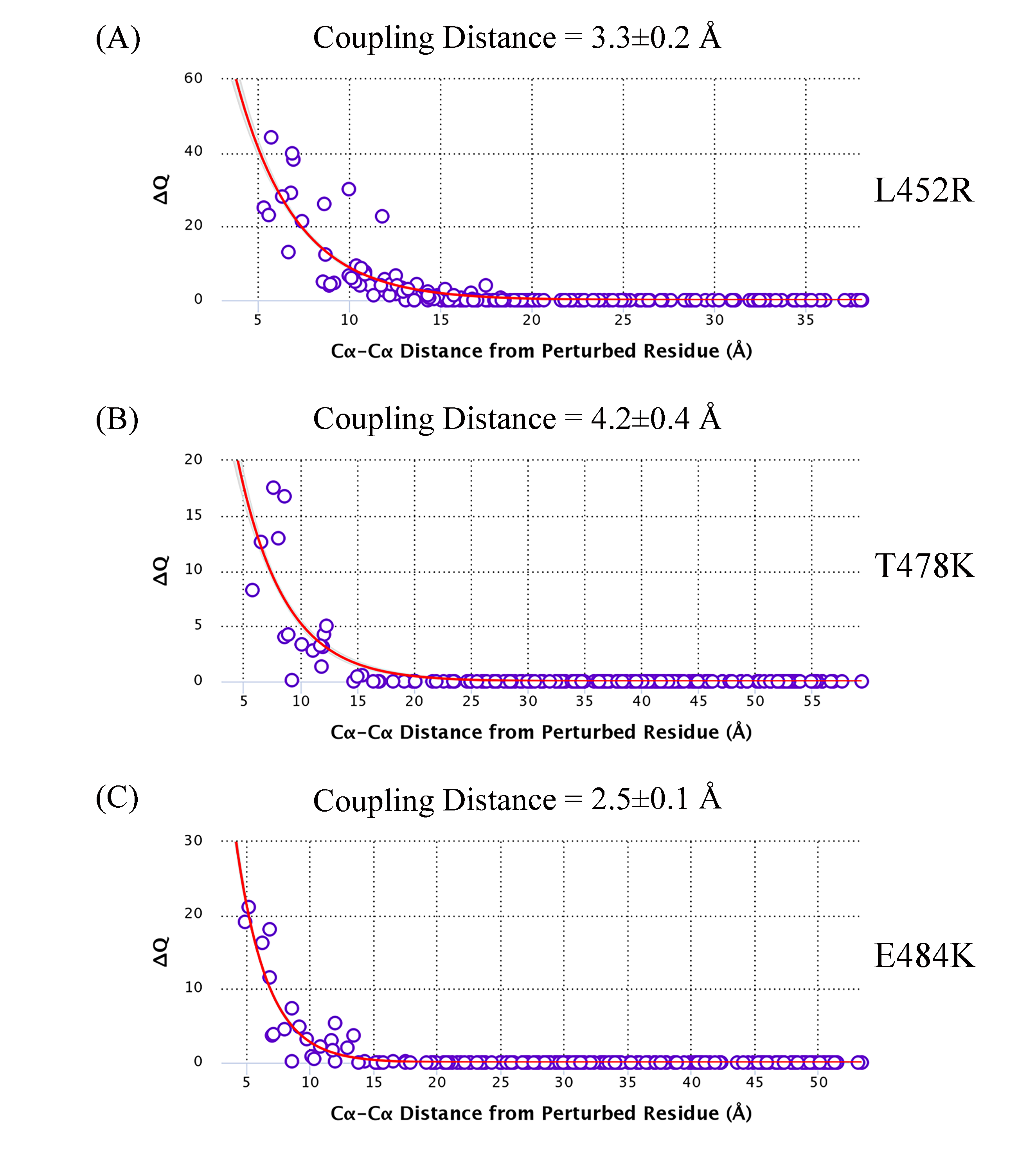

Supplement: Supplementary file 1 [file viruses-15-02056-s001.zip › Figure S9.tif]
